# Clinical, Epidemiological and Laboratory Features of Invasive *Candida parapsilosis* Complex Infections in a Brazilian Pediatric Reference Hospital during the COVID-19 Pandemic

**DOI:** 10.3390/jof9080844

**Published:** 2023-08-13

**Authors:** Paulo Henrique Peixoto, Maria Laína Silva, Fernando Victor Portela, Bruno da Silva, Edlâny Milanez, Denis de Oliveira, Aldaíza Ribeiro, Henrique de Almeida, Reginaldo Lima-Neto, Glaucia Morgana Guedes, Débora Castelo-Branco, Rossana Cordeiro

**Affiliations:** 1 Department of Pathology and Legal Medicine, Federal University of Ceará, Fortaleza 60430-160, Brazil; a59517c@gmail.com (P.H.P.); mlaina.silva@gmail.com (M.L.S.); portelafvm@gmail.com (F.V.P.); brunnonascimentodasilva@gmail.com (B.d.S.); edlanybio@gmail.com (E.M.); denisfgo@ufc.br (D.d.O.); glauciaguedes@ufc.br (G.M.G.); deb_castelobranco@yahoo.com (D.C.-B.); 2 Albert Sabin Children Hospital, Fortaleza 60410-794, Brazil; aldaizamr@gmail.com; 3 Department of Tropical Medicine, Federal University of Pernambuco, Recife 50670-901, Brazil; mrschinaski@gmail.com (H.d.A.); reginaldo.limant@ufpe.br (R.L.-N.)

**Keywords:** *Candida parapsilosis* complex, invasive candidiasis, immunocompromised patients, oncological patients

## Abstract

The present study aimed to describe the clinical, epidemiological and laboratory characteristics of invasive candidiasis by *C. parapsilosis* complex (CPC) in a Brazilian tertiary pediatric hospital during the COVID-19 pandemic. Clinical samples were processed in the BACT/ALERT^®^ 3D system or on agar plates. Definitive identification was achieved by MALDI-TOF MS. Antifungal susceptibility was initially analyzed by the VITEK 2 system (AST-YS08 card) and confirmed by the CLSI protocol. Patient data were collected from the medical records using a structured questionnaire. CPC was recovered from 124 patients over an 18-month period, as follows: *C. parapsilosis* (83.87%), *C. orthopsilosis* (13.71%) and *C. metapsilosis* (2.42%). Antifungal resistance was not detected. The age of the patients with invasive CPC infections ranged from <1 to 18 years, and most of them came from oncology-related sectors, as these patients were more affected by *C. parapsilosis*. *C. orthopsilosis* infections were significantly more prevalent in patients from critical care units. Invasive infections caused by different pathogens occurred in 75 patients up to 30 days after the recovery of CPC isolates. Overall, 23 (18.55%) patients died within 30 days of CPC diagnosis. Catheter removal and antifungal therapy were important measures to prevent mortality. COVID-19 coinfection was only detected in one patient.

## 1. Introduction

In the last decades, epidemiological studies have shown the dramatic worldwide increase in infections caused by non-albicans *Candida* species [1,2,3,4,5]. According to such studies, *C. parapsilosis* complex (CPC) are important agents of invasive candidiasis, becoming the second or third most prevalent pathogen, depending on the patient group and their geographic origin [6,7,8]. CPC species may be the second most common cause of candidemia in children [9,10] and have become widespread in some pediatric hospitals [11,12,13]. In general, *C. parapsilosis* sensu stricto is the most prevalent species, followed by *C. orthopsilosis* and *C. metapsilosis* [10,13,14].

*C. parapsilosis* sensu stricto and, possibly, *C. orthopsilosis* and *C. metapsilosis*, are commensal microorganisms of the human microbiota [15], but may act as opportunistic pathogens causing systemic infections in immunocompromised patients [16,17]. Accordingly, *C. parapsilosis* sensu stricto is frequently isolated from the hands, which makes it relevant in the context of hospital-related infections [2,18,19,20,21]. *C. parapsilosis* sensu stricto infections have been reported in hospital outbreaks [11,15,22] and associated with contaminated intravenous solutions or implanted devices, as well as the colonization of the hands of health-care workers with isolates from the hospital environment, followed by transmission to patients, and from the patients’ colonized skin to deep tissues [2,22,23].

Nearly two decades ago, *C. parapsilosis* sensu lato was recognized as a relevant pathogen of bloodstream infections in Brazilian pediatric hospitals [24,25]. Since then, studies have described regional differences regarding risk factors, underlying conditions and mortality rates in such a vulnerable population [9,26,27,28,29].

The COVID-19 pandemic brought worldwide healthcare challenges, including the management of opportunistic infections in hospitalized patients. Although children have less severe manifestations than adults [30], studies have described the significant impact of the lethality of COVID-19 in pediatric patients from low- and middle-income countries [31]. A nationwide database study conducted in Brazil showed that children and adolescents living in the poorest areas of the country (the Northeast and North regions) with previous comorbidities presented higher risk of death from COVID-19 [32].

The present study described the main characteristics of opportunistic invasive candidiasis caused by CPC species in a pediatric population with severe comorbidities admitted to a Brazilian tertiary referral hospital during 18 months throughout the COVID-19 pandemic.

## 2. Materials and Methods

### 2.1. Location

The study was conducted at Albert Sabin Children Hospital (HIAS), located in the city of Fortaleza (3°43′ S–38°32′ W), State of Ceará, northeastern Brazil. HIAS is a public reference institution for the care of children and adolescents. It has an emergency service with 65 beds (including 16 ICU beds), as well as a surgical center equipped with an ICU, where high and medium complexity interventions are performed in the areas of neurology, gastroenterology, urology, cardiology, orthopedics, oncology and plastic surgery. Each month, on average, 20,000 outpatient consultations, 30,000 laboratory exams, 650 hospitalizations and 350 surgeries are performed at HIAS. The hospital also has a Pediatric Cancer Center, a reference unit for the diagnosis and treatment of childhood cancers in the North and Northeast regions of Brazil. This unit has approximately 500 patients, providing an annual average of 12,500 outpatient visits and 6700 chemotherapy sessions. HIAS has a Hospital Infection Control Committee, whose activities are in line with the Brazilian government’s National Program for Prevention and Control of Healthcare-Associated Infections.

The State of Ceará has an estimated population of 9,240,580; its capital, Fortaleza, has the highest population density among all Brazilian capitals—7,786,044 inhabitants per km [33]. According to official data, Ceará has a Gini coefficient of 0.56, and nearly half of its inhabitants lives with less than 50% of the minimum wage. Nearly 18% have daily earnings below US$ 1.9 and are regarded as people living in extreme poverty by the World Bank. The economy is roughly based on agriculture (5%), industry (18%) and services (78%) [33]. The tropical semiarid climate comprises 80% of the state’s area; the climate in the city of Fortaleza is classified as tropical sub-humid in the coastal region [34].

### 2.2. Study Design, Ethical Approval and Definitions

This is a single center prospective cross-sectional study. Approval was obtained by the institutional ethics committee (number 4.207.133), and written informed consent was obtained from the parents of the participants. Patients at 0 to 18 years of age, hospitalized from 11 August 2020 to 11 February 2022, who presented invasive CPC candidiasis were included in the study. Laboratory exclusion criteria, including inadequate collection, transport period and temperature, were applied in this study.

Candidemia caused by CPC was defined as the isolation of any CPC species from blood culture obtained from peripheral vein or intravascular catheter from symptomatic patients with no signs of infection in other body sites. Invasive candidiasis was defined as the isolation of any CPC species from organ biopsies, cerebrospinal fluid (CSF), cavitary fluids (peritoneal, pleural, pericardial and synovial fluids), urine and respiratory secretions in a symptomatic patient. Candidemia occurring more than 30 days after the first recovery of CPC was defined as a new case. Healthcare-associated infections were defined as those occurring 48 h after hospital admission. Patients were tested for COVID-19 upon admission to hospital. All patients were tested for SARS-CoV-2 by RT-PCR or rapid antigen test using nasopharyngeal swabs.

### 2.3. Data Collection

Clinical-epidemiological data were collected from the medical records using a structured questionnaire comprising closed-ended questions. The main variables studied were related to (i) patient identification (gender, age and weight at admission); (ii) hospital admission (date and reason for hospitalization, hospital care sector, blood work, signs and symptoms); (iii) risk factors (immunosuppression, transplantation, prematurity, antimicrobials, chemotherapy, parenteral nutrition, medical devices and previous surgeries), and comorbidities; (iv) microbiological findings (bacterial co-isolation); (v) fungemia (clinical characteristics, signs and symptoms, treatment and outcome).

### 2.4. Laboratory Procedures

Clinical samples (blood, CSF and cavitary fluids) were processed using the BACT/ALERT^®^ 3D system (bioMérieux, Craponne, France). Urine samples were manually streaked on CHROMagar^TM^ Orientation plates (Plastlabor, Rio de Janeiro, Brazil) using a calibrated 0.001-mL loop. Culture growth of 10^5^ cfu/mL or greater from symptomatic inpatients were considered positive [35]. Respiratory samples were processed on MacConkey agar (Plastlabor, Brazil) and Chocolate agar (Plastlabor, Brazil) plates. Catheter tips were processed by the semi-quantitative Maki technique on Columbia agar plate supplemented with 5% sheep blood; cultures exhibiting growth ≥15 cfu were considered positive [36]. No fungal biomarkers were evaluated in this study.

Suspected *Candida* spp. colonies were streaked on CHROMagar *Candida* plates and further analyzed by VITEK^®^ 2 Compact system (bioMérieux, France). Definite identification was achieved by MALDI-TOF MS (Autoflex III, Bruker Daltonics Inc., Billerica, MA, USA/Bremen, Germany). Obtained spectra were compared with the BiotyperTM version 3.1 database (Bruker Daltonics, Germany/USA) for yeast identification, considering the identification with score values ≥2 [37].

Antifungal susceptibility was performed with VITEK 2 AST-YS08 antifungal susceptibility card (bioMérieux^®^, France). Obtained results were confirmed by microdilution, according to M27-A3 guidelines [38]. Strains were tested for susceptibility to amphotericin B (AMB, ≤0.25 to >16 µg/mL), fluconazole (FLC, ≤0.5 to >64 µg/mL), voriconazole (VRZ, ≤0.125 to >8 µg/mL), caspofungin (CAS, ≤0.125 to >8 µg/mL) and micafungin (MIC, ≤0.06 to >8 µg/mL) [38].

### 2.5. Biofilm Formation

Biofilms were formed as described by [39], with adaptations. Each clinical isolate was previously grown on potato dextrose agar for 48 h at 35 °C. After this period, the colonies were suspended in sterile 0.9% saline, and the turbidity was adjusted to 1 × 10^6^ cfu/mL in RPMI-1640 RPMI (Sigma-Aldrich, Burlington, MO, USA) and buffered to pH 7.0 with 0.165 M morpholinepropanesulfonic acid (MOPS; Sigma-Aldrich, MO, USA); 200 µL of each suspension were transferred to flat 96-well polystyrene plates and incubated at 35 °C for 48 h. After that, biofilm biomass was analyzed by the crystal violet staining technique [40]. Biofilm production was classified according to Stepanovic et al. [41].

### 2.6. Statistical Analysis

Categorical data were expressed in absolute frequencies and percentages, and the analyses of these data were performed using the Chi-square or Fisher’s exact test. Software R (version 4.2.1) was used, and a significance level of 5% (*p* < 0.05) was considered for all analyses.

## 3. Results

### 3.1. Patient Demographic, Clinical Characteristics and Outcomes

Of the 124 patients enrolled in this study, 68 were male (54.83%; 68/124) and 56 were female (45.16; 45/124). The majority of infections occurred in patients between two and 12 years of age (49.19%; 61/124); CPC infections occurred at similar frequencies among patients under two years old (25%; 31/124) and those between 12 to 18 years old (25.8%; 32/124). Patients were mainly admitted to oncology wards (62.9%; 78/124), and *C. parapsilosis* sensu stricto infections were significantly higher in these individuals (68.26%; 71/104) (*p* = 0.012). *C. orthopsilosis* infections were significantly more frequent (52.94%; 9/17) (*p* = 0.012) in patients admitted to critical care units (intensive care units and emergency rooms). Duration of hospitalization (mean ± SD) was 47.01 ± 37.88 days.

As expected, cancer was the main individual underlying condition/comorbidity in patients with CPC infections (60.48%; 75/124); 68.35% (68/104) of all *C. parapsilosis* infections occurred in patients that presented cancer as the only reported underlying condition (*p* = 0.023). CPC infections also occurred in patients who presented more than one underlying condition (16.12%; 20/124). Oncologic patients infected with CPC mainly presented acute lymphoblastic leukemia (44%; 33/75), acute myeloid leukemia (12%; 9/75) and Ewing sarcoma (5.33%; 4/75) as an underlying disease.

The main risk factors detected for CPC infections included central venous catheterization (92.74%; 115/124) and other implanted devices (29.83%; 37/124), immunosuppressive therapy (70.96%; 88/124), neutropenia (34.97%; 43/124) and previous antimicrobial polytherapy (79.03%; 98/124).

The management of the majority of infected patients included catheter removal and antifungal therapy (54.03%, 67/124), antifungals alone (28.22%; 35/124) or device removal alone (8.06%; 10/124). Therapeutic data regarding 12 patients were not available on medical records. Patients were treated with amphotericin B deoxycholate (single dose of 1.0–1.5 mg/kg/day), fluconazole (8 to 12 mg/kg/day) or micafungin (10 mg/kg/day for neonates, 3 mg/kg/day for children ≤30 kg and 2.5 mg/kg/day for children ≥30 kg). Duration of antifungal therapy varied from 1 to 14 days. Most patients received antifungals for 2 weeks after the diagnosis of candidemia. Antifungal therapy was suspended after clearance of *Candida* from the bloodstream and/or resolution of symptoms and signs attributable to candidemia/deep-seated infection. Antifungal therapy was changed in three patients: from micafungin to amphotericin B, from fluconazole to micafungin, and from fluconazole to amphotericin B. Further details regarding such alterations were not available on medical records.

A total of 94 episodes (75.8%; 94/124) were considered healthcare-associated infections; CPC candidemia accounted for 88 cases (70.96%; 88/124), and six (4.83%; 6/124) were considered other invasive candidiasis episodes. Despite catheter removal and antifungal therapy, population mortality reached nearly 19% (18.54; 23/124) of the patients.

Details regarding epidemiological characteristics of the studied patients are shown in Table 1.

### 3.2. Candida parapsilosis Complex Isolates and Antifungal Susceptibility

Invasive infection was diagnosed in 124 patients. Isolates were recovered mainly from catheter-drawn blood (44.35%; 55/124) and peripheral blood (37.09%; 46/124) (Table 2). *C. parapsilosis* sensu stricto (from now on *C. parapsilosis*) accounted for the majority of the isolates (n = 104, 83.87%), followed by *C. orthopsilosis* (n = 17, 13.71%) and *C. metapsilosis* (n = 3, 2.42%). Antifungal resistance was not detected among the isolates.

CPC was the only pathogen in 106 cases (85.48%; 106/124). However, concomitant bacteremia (1.61%; 2/124) and co-infections were also detected in the study (4.83%; 6/124). Co-infection with SARS-CoV-2 during CPC candidemia only occurred in one patient (Table 3). Bacterial pathogens were the most frequent infectious agent recovery before CPC candidemia. After the diagnosis of CPC infection, a total of 75 patients showed deep infections, mainly by one bacteria species (16%; 12/75) or more than one bacterial pathogen (46.66%; 35/75); infections caused by *Candida* spp. accounted for 37.33% of such cases (28/75). CPC isolates were detected in up to 30 days after diagnosing incident candidemia/deep-seated infection (*C. parapsilosis*, n = 21; *C. orthopsilosis*, n = 4) from blood cultures obtained from a peripheral vein or intravascular catheter. One patient showed concomitant infection with *C. parapsilosis* and SARS-CoV-2 after the diagnosis of candidemia. Details regarding microbial isolates are shown Table S1.

Most CPC isolates were biofilm-producers (91.93%; 114/124). Of these, 90 were strong-biofilm producers (78.94%; 90/114), 18 moderate biofilm-producers (15.78; 18/114) and six weak biofilm producers (5.26; 6/114). *C. parapsilosis* isolates were significantly associated with the strong-biofilm producer phenotype (*p* = 0.03); among such isolates, those recovered from catheter-drawn blood were more likely to be strong-biofilm producers (*p* = 0.01).

## 4. Discussion

Candidemia is an important opportunistic infection in pediatric populations at risk, mainly neonates and those with hematologic malignancies [42] and patients in mechanical ventilatory support and immunosuppressive therapy [43]. However, risk factors for candidemia may vary according to patient populations and fungal etiology, which brings challenges to clinical diagnosis [44]. Accordingly, epidemiological studies help to understand the infectious landscape and, therefore, to predict outcomes.

In this study, CPC isolates were identified by MALDI-TOF—a rapid, accurate, and cost-effective method to assess these cryptic species [37], reliable for routine laboratory tests. Identification of CPC species can also be performed by sequencing of the internal transcribed spacer (ITS) region and D1/D2 domain of the 26S rRNA gene [23], as well as by amplification of the gene encoding secondary alcohol dehydrogenase enzyme, followed by analysis of the presence of *Ban*I restriction site [5,13]. However, it is important to note that, at the present time, proper identification of CPC species cannot be achieved by the commercial systems available, such as API 20C AUX and Vitek 2 YST ID Card.

CPC has been pointed as an important pathogen of invasive infections in pediatric patients [9,10,27,28,29,45]. In the present study, invasive CPC candidiasis occurred in 124 pediatric patients over a period of 18 months. The high number of CPC episodes during this research caught our attention. The prevalence of invasive CPC candidiasis varies according to region, and nationwide multicentric data may not be available for many countries. A recent study conducted with retrospective data of candidemia over 14 years in Turkey revealed *C. parapsilosis* sensu lato as the most commonly isolated species, with a total of 148 isolates [46]. Liu et al. [47] described 12 cases of CPC candidemia over a period of 11 years in a Canadian tertiary care pediatric hospital. However, CPC has been recognized as the second most isolated pathogen of invasive infections in Southern European hospitals (Portugal, Spain, Italy and Greece), whereas in United Kingdom, Sweden and Denmark, CPC ranks in third position [48]. Studies conducted in developing countries also described a high number of CPC isolates [12,49]. Worldwide changes in the epidemiology of candidemia over the last decades have been widely reported, and the prominence of CPC species, mainly in developing countries, is remarkable.

Although the global emergence of fluconazole-resistant *C. parapsilosis* lato sensu is a matter of concern [22], all the strains described herein were susceptible to azoles. It is important to emphasize, however, that continuous prophylaxis with fluconazole may lead to the selection of resistant strains at some point in the future. Previous studies conducted in Brazil have shown the presence of azole-resistant strains of *C. parapsilosis* in critically ill patients associated with high mortality rates [27,50,51,52,53]. According to Daneshnia et al. [22], worldwide fluconazole resistance in *C. parapsilosis* sharply increased during the COVID-19 pandemic. This scenario demonstrates the need for constant antifungal resistance monitoring studies in order to implement measures to prevent the increase of resistance in *C. parapsilosis* isolates in Brazilian hospitals.

Many experts do not recommend drawing blood for blood culture from catheters due to the risk of device colonization with skin microbiota. However, due to practical reasons, such as reducing patient discomfort and the impossibility of obtaining more than one sample, catheter-drawn blood samples were frequently enrolled in this study. Although skin contamination may be a relevant issue for the diagnosis of candidemia, classifying an isolate as a contaminant is a difficult task. In the present study, skin contamination was suspected when typical contaminants were found: *Bacillus* spp., *Corynebacterium* spp. and *Propionibacterium acnes*. In these cases, such results were ruled out, and another sample collection was performed. Therefore, positive blood cultures for any CPC species in a symptomatic patient using catheters and/or other implanted devices was considered a case of candidemia.

Pediatric candidemia is complicated when mixed infections occur, as children may present with a longer duration of septic symptoms and a relatively higher risk of candidemia attributable mortality [54]. In the present study, concomitant bacteremia and co-infections were seen in 12.9% of the patients. The identified pathogens were typical bacterial pathogens of healthcare-associated infections, most of them with worrying antibiotic resistance, including *Pseudomonas* spp., coagulase-negative *Staphylococcus*, *Klebsiella* spp., *Acinetobacter* spp. and *Enterococcus*. Pediatricians should be aware of the clinical impacts of such mixed infections, as they affect patient outcome. Some patients also showed deep-seated microbial infections before CPC isolation, and this could have influenced the development of candidemia, as the antibiotic treatment itself is a well-recognized risk factor for fungal infections.

Nearly 92% of CPC isolates described in this study were able to produce biofilms. This result has a great importance in understanding the physiopathology of invasive candidiasis. These species are prone to form biofilms on catheters, becoming more resistant to host immune response and antifungal therapy; sessile cells can also detach from catheters and invade the bloodstream [2,19]. It is noteworthy that all patients included in this study were in use of catheters and/or other implanted devices during candidemia or deep-seated CPC episode.

The majority of invasive infections described herein occurred in oncologic patients, mainly those with acute lymphoblastic leukemia and acute myeloid leukemia. Hematologic malignancies are important risk factors for candidemia in children [42,55], either due to the primary neoplastic disease itself, changes in mucosal integrity and function or the anticancer immunosuppressive therapy. In addition, the oncologic patients enrolled in the present study received prophylactic fluconazole and had multiple catheters inserted, ultimately making them more vulnerable to opportunistic invasive infections. In fact, CPC candidemia has been widely described in such a vulnerable population [11,55,56].

The relevance of *Candida* spp. as agents of respiratory infections has been a matter of debate. Indeed, after 48h of mechanical ventilation, up to 20% of patients are colonized with *Candida* at the tracheobronchial site, and *Candida* can be found in tracheal aspirate, even in healthy adults [57]. Previous studies have linked respiratory tract *Candida* colonization with worse clinical outcomes [58]. In addition, mechanically ventilated patients colonized with *Candida* were more at risk to develop *Pseudomonas aeruginosa* pneumonia [59]. Schnabel and colleagues [57] argued that *Candida* pneumonia exists as a rare clinical entity, but its diagnosis is challenging. Lung biopsy is the most reliable method [57], but it is not feasible most of the time, due to the risk of thrombocytopenia and coagulopathy. Measuring (1, 3)-β-D-glucan in respiratory samples could be a less invasive biomarker of *Candida* pneumonia [60].

In the present study, *C. parapsilosis* was recovered from the tracheal aspirate of three patients (Id.32; Id. 45; and Id. 89) admitted to critical care units. These results were included in our research, as medical staff considered them a significant indication of *Candida* infection. These patients were at different ages (2 years old, 14 years old and 2 months old, respectively) and had been admitted to ICU for long periods (47 days, 145 days and 58 days, respectively). They were in use of CVC and other devices and also were exposed to antibiotic therapy (meropenem and linezolid, meropenem, amikacyn, teicoplanin, polymyxin B and cefepime, respectively). All of them were suspected of having sepsis; the older patient was initially admitted for presenting signs of encephalitis and subsequently died of multiple organ failure. After the recovery of *C. parapsilosis* from tracheal samples, due to the risk of candidemia/deep-seated infection, antifungal therapy was initiated with amphotericin B or micafungin.

While urinary tract infections are some of the most common microbial infections in both hospital and community settings, the finding of *Candida* species in urine represents a clinical challenge for physicians, as it may be related to contamination, colonization, urinary tract infection or invasive candidiasis. In the present study, a total of four isolates were recovered from urine, all of them considered an indication of CPC infection (Id.8 and Id. 116, *C. parapsilosis*; Id. 129 and Id.132, *C. orthopsilosis*). These were symptomatic patients admitted to critical care units for variable periods of time (up to 202 days) with multiple risk factors for invasive candidiasis (use of CVC, previous antibiotic therapy and underlying diseases). Isolates were recovered from both urine and blood samples on the same day (Id. 129) or only from urine (Id. 8, Id. 116, Id. 132). Patients were treated with amphotericin B or azoles, and death was registered in one patient (Id. 129). Although CPC may be found as colonizers of skin and mucous membranes [15], isolates from non-bloodstream sources should receive attention, as they are prone to cause invasive disease in high-risk patients [16,17].

Although eight patients were hospitalized with suspected COVID-19 infection, only one of them yielded positive viral detection. COVID-19 is usually less severe in children than in adults [30]. However, analysis of thousands of cases in Brazil revealed that malignancies, heart diseases, genetic diseases and neurological disorders are important risk factors for death among hospitalized children and adolescents with COVID-19 [31], most of which were also observed in the patients with CPC infection described herein. Authors also reinforced that disparities in health care and poverty also contribute to the burden of COVID-19 in such patients [32]. Although the studied population described herein came from a public health institution designed to provide healthcare assistance to socioeconomically disadvantaged children and adolescents, the low incidence of COVID-19 in these patients could be related to social distancing and other preventive measures adopted by their parents during the pandemic.

## 5. Conclusions

This study described the importance of invasive candidiasis by *C. parapsilosis* complex in pediatric patients in Brazil, especially those with malignancies. Given the global emergence of fluconazole-resistant CPC, as well as due to the use of this antifungal as a prophylactic agent in high-risk patients in Brazil, azole resistance in *Candida* spp. should be continuously monitored. Infection control measures (i.e., compliance with staff hand hygiene protocols, avoidance of unnecessary invasive devices and broad-spectrum antibiotics, removal of catheters in patients with candidemia, etc.) must be prioritized. Clinicians should be aware that invasive candidiasis by CPC can occur concomitantly with bacteremia or other co-infections, amplifying the burden of fungal disease in such vulnerable populations. COVID-19 infection, however, was rare among our patients.

## Figures and Tables

**Table 1:** The main clinical and epidemiological features of patients with invasive *Candida parapsilosis* complex infection (n = 124).

Variables	*C. parapsilosis* (n = 104)	*C. orthopsilosis* (n = 17)	*C. metapsilosis* (n = 3)
**Gender**			
Male	60	8	0
Female	44	9	3
**Age group (years)**			
<2	23	7	1
2 to 12	55	4	2
12 to 18	26	6	0
**Hospital Ward**			
Oncology	71 *	6	1
Critical care unit	20	9 *	2
Hospital wards	13	2	0
**Underlying conditions/Comorbidities**			
Cancer	68 *	6	1
Acute lymphoblastic leukaemia	29	3	1
Acute myeloid leukaemia	9	0	0
Ewing sarcoma	4	0	0
Hydrocephalus	3	3	0
Genetic disorders	2	3	0
Heart conditions	3	0	0
Gastrointestinal diseases	2	0	0
Encephalopathy	2	0	0
Bone disease	0	1	0
More than one condition/disease	15	3	2
Others	2	0	0
**Risk factors**			
Central venous catheter			
PICC ^a^	76	14	2
Double lumen	38	10	2
Other devices			
Nasoenteral feeding tube	25	6	1
Nasogastric feeding tube	22	8	1
Indwelling bladder catheter	23	5	2
Intravenous catheter	20	9	0
Orotracheal tube	21	6	2
Orogastric feeding tube	8	5	0
Ventriculoperitoneal shunt	7	4	0
Tracheostomy tube	7	2	0
Parenteral nutrition	9	1	1
Immunosuppressive therapy			
Corticosteroids, antineoplastics and other medications concomitantly	68	6	2
Corticosteroids and antineoplastics concomitantly	39	5	0
Antineoplastics	23	1	1
Corticosteroids	14	4	1
Neutropenia	40	3	0
Prior antimicrobial polytherapy ^b^	82	14	2
Prior antibiotic therapy ^c^	93	15	3
Previous antifungal therapy ^c^	46	2	2
Concomitant bacteremia ^d^	2	0	0
Concomitant deep-seated infection ^d^	1	0	0
Co-infections ^d^	5	0	0
Co-infection with COVID-19 ^d^	1	0	0
**Management**			
Antifungal therapy	30	4	1
Amphotericin B	38	3	0
Fluconazole	21	5	2
Micafungin	26	4	0
Combined antifungal therapy			
Amphotericin B + Micafungin	1	0	0
Fluconazole + Micafungin	0	1	0
Amphotericin B + Fluconazole	1	0	0
Catheter removal	7	2	0
Catheter removal and antifungal therapy	57	9	1
Indwelling bladder catheter removal	0	1	0
Not informed	10	1	1
**Health-care associated infections**	80	13	1
Candidemia	76	11	1
Other invasive candidiasis manifestations	4	2	0
**Outcome**			
Discharge	82	12	2
Death	19	4	0
Transfer to outpatient facilities	3	0	0
Transfer to another hospital	0	1	1

* *p* < 0.05; ^a^ Peripherally inserted central catheter; ^b^ Antibiotics, antifungals and antivirals up to 48 h of CPC isolation. ^c^ Administered up to 48 h of CPC isolation. ^d^ Healthcare-associated infections, as indicated by the institutional Healthcare Infection Prevention and Control Committee.

**Table 2:** Frequency of *Candida parapsilosis* complex members in clinical samples from patients with invasive infection.

Clinical Specimen	*C. parapsilosis * (n = 104)	*C. orthopsilosis* (n = 17)	*C. metapsilosis* (n = 3)
Blood culture	84	14	3
Tracheal aspirate	3	0	0
Catheter-tip	3	0	0
Urine	1	2	0
Peritoneal fluid	1	0	0
Cerebrospinal fluid	0	1	0
More than one clinical specimen	12	0	0

**Table 3:** Additional bacterial and fungal pathogens isolated during *Candida parapsilosis* complex invasive infections.

Isolated Microrganisms	*C. parapsilosis* (n = 104)	*C. orthopsilosis* (n = 17)	*C. metapsilosis* (n = 3)
**Concomitant bacteremia**			
* Pseudomonas putida* ^A^	1	0	0
* Staphylococcus epidermidis* ^B^	1	0	0
**Concomitant infection** ^**¥**^			
* Acinetobacter baumannii* ^C^	1	0	0
**Co-infections** ^**ƛ**^			
* P. aeruginosa* ^C^	1	0	0
* Klebsiella pneumoniae* spp. *Pneumonia* ^C^	1	0	0
* S. epidermidis* ^D^	1	0	0
* S. epidermidis* ^D^	1	0	0
* C. glabrata* ^E^	1	0	0
SARS-CoV-2 ^F^	1	0	0

^¥^ Isolates recovered from the same deep-seated clinical specimen other than blood. ^ƛ^ Isolates recovered from a different deep-seated clinical specimen other than blood. ^A^: Peripherally inserted central catheter. ^B^: Central venous catheter (double lumen). ^C^: Tracheal aspirate < 10^6^ cfu/mL. ^D^: Catheter-drawn blood. ^E^: Urine < 10^6^ cfu/mL. ^F^: Nasopharyngeal swab.

## Data Availability

Not applicable.

## Supplementary Materials

The following supporting information can be downloaded at: https://www.mdpi.com/article/10.3390/jof9080844/s1, Table S1. Microbiological characteristic of healthcare-associated infections occurred before or after invasive candidemia by CPC.
