# Supplementary material for: Clinical, Epidemiological and Laboratory Features of Invasive Candida parapsilosis Complex Infections in a Brazilian Pediatric Reference Hospital during the COVID-19 Pandemic
Source: J Fungi (Basel). 2023 Aug 13;9(8):844. doi: 10.3390/jof9080844 (PMC10456047; doi:10.3390/jof9080844)
Supplement: Supplementary file 1 [file jof-09-00844-s001.zip › jof-2455694-supplementary.pdf]

Table S1. Microbiological characteristic of healthcare-associated infections occurred before or after invasive candidemia by CPC.

Isolated microrganisms	*C. parapsilosis* (n=104)	*C. orthopsilosis* (n=17)	*C. metapsilosis* (n=3)
**BEFORE CPC CANDIDEMIA[Φ]**			
Enterobacter cloacae complex[B]	1	0	0
Escherichia coli[C]	1	0	0
Proteus mirabilis[C]	1	0	0
S. capitis[B]	1	0	0
S. epidermidis[B]	1	0	0
S. hominis spp. *hominis*[D]	1	0	0
S. warneri[D]	1	0	0
C. parapsilosis complex[D]	0	1	0
C. tropicalis[B]	1	0	0
***Polymicrobial infections*[μ]**			
C. parapsilosis complex[B] and *S. warneri*[B]; *Elizabethkingia meningoseptica*[F]; *K. pneumoniae*[B]; *S. epidermidis*[D] (2x); and coagulase-negative *Staphylococcus* spp.[D]	1	0	0
K. pneumoniae[A]; and *P. aeruginosa*[A]; *C. tropicalis*[D]; and *S. epidermidis*[D]	1	0	0
C. parapsilosis complex[B, D, F]; and *K. pneumoniae*[B]	1	0	0
K. pneumoniae[B, D]; and *P. aeruginosa*[C]	1	0	0
C. albicans[D]; and *S. epidermidis*[B]	1	0	0
E. cloacae complex[B]; and *P. aeruginosa*[A]	1	0	0
**AFTER CPC CANDIDEMIA[£]**			
C. parapsilosis complex[B, C]	21	4	0
K. pneumoniae[B, C] (3x and 2x)	4	0	0
E. coli[C]	1	0	0
S. epidermidis[B]	1	0	0
***Polymicrobial infections*[μ]**			
K. pneumoniae[A] and *P. aeruginosa*[A]; *C. parapsilosis* complex[B]; *K. oxytoca*[G]; *P. aeruginosa*[B]; and *S. epidermidis*[B, D]	1	0	0
C. parapsilosis complex[B]; *C. tropicalis*[B,D] and *K. pneumoniae*[D]	1	0	0
K. pneumoniae[D]; and *P. aeruginosa*[D] (2x); *C. parapsilosis* complex[D]; and *K. pneumoniae*[D] (2x)	1	0	0
C. parapsilosis complex[B,D]; *S. epidermidis*[D]; and *Stenotrophomonas maltophilia*[C]	1	0	0
C. glabrata[C]; and *C. parapsilosis* complex[B, C, E]	1	0	0
C. parapsilosis complex[D]; and *Pseudomonas* spp.[C] (> 10^6 cfu/mL)	1	0	0
C. parapsilosis complex[D] (3x); and SARS-CoV-2[E]	1	0	0

C. parapsilosis complex[D] and *S. hominis* spp. *hominis*[B]	1	0	0
K. pneumoniae[C]; and *P. aeruginosa*[C] (2x)	1	0	0

[Φ] Isolates recovered at any time during hospitalization, before the diagnosis of CPC candidemia / deep-seated infection

[μ] Isolates recovered on the same day

[£] Isolates recovered up to 30 days after incident CPC candidemia / deep-seated infection

[A C]: Tracheal aspirate < 10^6 cfu/mL
[B D]: Catheter-drawn blood
[D E]: Peripheral blood
[C F]: Urine < 10^6 cfu/mL
[E G]: Nasopharyngeal swab
[F H]: Catheter tip
[G I]: Rectal swab
